# Whole-Body Cryotherapy Alters Circulating MicroRNA Profile in Postmenopausal Women

**DOI:** 10.3390/jcm12165265

**Published:** 2023-08-12

**Authors:** Magdalena Wiecek, Justyna Kusmierczyk, Jadwiga Szymura, Grzegorz Kreiner, Zbigniew Szygula

**Affiliations:** 1 Department of Physiology and Biochemistry, Faculty of Physical Education and Sport, University of Physical Education in Kraków, 31-571 Kraków, Poland; justyna.kusmierczyk@awf.krakow.pl; 2 Department of Clinical Rehabilitation, Faculty of Motor Rehabilitation, University of Physical Education in Kraków, 31-571 Kraków, Poland; jadwiga.szymura@awf.krakow.pl; 3 Department Brain Biochemistry, Maj Institute of Pharmacology, Polish Academy of Sciences, 31-343 Kraków, Poland; kreiner@if-pan.krakow.pl; 4 Department of Sports Medicine and Human Nutrition, Institute of Biomedical Sciences, Faculty of Physical Education and Sport, University of Physical Education in Kraków, 31-571 Kraków, Poland; zbigniew.szygula@awf.krakow.pl

**Keywords:** microRNAs expression, whole-body cryotherapy, metabolic syndrome, postmenopausal women, metabolism regulation, visceral obesity

## Abstract

The incidence of metabolic syndrome (MetS) increases with age, especially in women. The role of microRNAs (miRs) in the regulation of metabolism is postulated. The aim of the study is to identify miRs that may be markers of MetS and to assess changes in miRs expression as a result of 10 and 20 whole-body cryotherapy treatments (WBC; 3 min, −120 °C) in postmenopausal women with MetS (M-60, BMI 30.56 ± 5.38 kg/m^2^), compared to healthy postmenopausal (H-60, BMI 25.57 ± 2.46 kg/m^2^) and healthy young women (H-20, BMI 22.90 ± 3.19 kg/m^2^). In a fasting state, before 1 WBC and after 10 WBCs, as well as 20 WBCs, the expression of miR-15a-5p, miR-21-5p, miR-23a-3p, miR-146a-5p, miR-197-3p, miR-223-3p, fasting blood glucose (FBG) and blood lipid profile were determined. miR-15a-5p and miR-21-5p were down-regulated in M-60, while miR-23a-3p and miR-197-3p were up-regulated, and miR-223-3p down-regulated in M-60 and H-60, compared to H-20. Significant positive correlations between up-regulated (mostly for miR-23-3p and miR-197-3p) and significant negative correlations between down-regulated (mostly for miR-15a-5p) miRs and markers of body composition as well as metabolic disorders were observed. After 20 WBCs, miR-15a-5p expression was up-regulated in all groups. In H-60, down-regulation of miR-197-3p expression occurred after 10 WBCs and 20 WBCs. Following 10 WBCs, FBG decreased in all groups, which intensified in M-60 post-20 WBCs. In our research, it has been shown that miR-23a-3p and miR-197-3p are accurate markers of MetS and MetS risk factors, while miR-15a-5p and miR-23a-3p are precise markers of body composition disorders. WBC is an effective treatment for up-regulating miR-15a-5p and lowering glucose levels in young and postmenopausal women and down-regulating miR-197-3p expression in postmenopausal women. It may be an adjunctive effective treatment method in MetS and hyperglycemia.

## 1. Introduction

The coexistence of body composition and atherogenic lipid profile disorders, hyperglycemia and hypertension, according to the diagnostic criteria of the National Cholesterol Education Program—Adult Treatment Panel III (NCEP—ATP III), give rise to the diagnosis of metabolic syndrome (MetS). The most common disorder in MetS is visceral obesity with concomitant low-grade chronic inflammation [1,2].

In research carried out among a general population from 10 European countries, covering approximately 70,000 people aged 19–78, it was found that MetS occurs in 19.9% of men and 32.1% of women (NCEP—ATP III criteria) [3]. The incidence of MetS increases with age, reaching 10.5% and 21.8% in men and 7.6% and 37.6% in women aged 19–39 and 60–78 years, respectively [3].

People with MetS are at a higher risk of developing cardiovascular diseases (CVD), insulin resistance (IR) and type-2 diabetes (T2DM) [1,2]. Although in a global study covering 195 countries it was concluded that the overall incidence and mortality due to CVD significantly decreased in the years 1990–2017, and the ratio of mortality to morbidity was stable, CVD mortality remains a global problem [4]. In 2017, CVD led to 17.8 million deaths worldwide, and it is predicted that in 2030 this number may increase to 23.3 million and will remain the leading cause of death [5,6,7]. Increased arterial stiffness in people with MetS may be a preclinical predictor of cardiovascular dysfunction and development of CVD, connected with lipid profile disorders and increased triglyceride-glucose index (TyG) [8].

Due to the very high incidence of MetS and the risk of related complications, new models of non-pharmacological therapeutic procedures with scientifically proven effectiveness should be implemented as additional measures in the treatment of patients with MetS, apart from increasing physical activity [9,10] and modifying diet [11,12]. The results of research on the subject indicate that such a method may be whole-body cryotherapy (WBC). The mechanisms regarding the metabolic impact of cryogenic temperatures have not been explained so far [13,14,15,16].

WBC is based on short-term (1–3 min), repeated (every day or every other day) action of cryogenic temperatures (from −110 °C to −160 °C) on the largest possible surface of exposed skin. As a result of cryogenic temperature, cutaneous blood vessels narrow and blood flow is limited, followed by reperfusion [16,17,18].

It has been proven that after applying 20 WBC treatments, the concentration of total cholesterol (T-CHOL), triglycerides (TG) and low-density lipoprotein cholesterol (LDL-C) begins to decrease, while the concentration of high-density lipoprotein cholesterol increases (HDL-C) [19]. Beneficial changes in the concentration of some peptide hormones regulating carbohydrate and lipid metabolism under the influence of cryogenic temperatures were also found [20,21]. When WBC was used before an hour of physical training, already after 10 treatments, beneficial changes in lipid profile were obtained, i.e., the reduction of T-CHOL, LDL-C and TG levels, the reduction of oxidative stress and the reduction in markers of endothelial inflammation [22,23].

The regulation of metabolic processes in response to endogenous and environmental stimuli requires the involvement of many controlling mechanisms. Currently, the role of microRNAs (miRNAs or miRs) in the regulation of metabolism, insulin signaling pathways, adipocytokine synthesis, as well as in the regulation of inflammatory processes, is being increasingly recommended [2,24,25,26]. The expression of some miRs is altered by exposure to cold [27].

MicroRNAs are endogenous, short-chain, non-coding RNAs that play an important role in gene regulation among humans, complementarily being linked to mRNAs of protein-coding genes to direct their post-transcriptional repression [28]. Some miRNAs correlate with disorders characteristic of MetS [29,30]. Thus, the profile of miRNA changes can be postulated as a potential biomarker for diagnosis, prognosis or assessment of disease progression, but also the effects of therapy [30,31].

In our study, we selected six different miRNAs (miR-15a-5p, miR-21-5p, miR-23-3p, miR-146a-5p, miR-197-3p, miR-223-3p) involved in the regulation of processes that are the basis of metabolic and cardiovascular disorders and may play a significant part in the development of MetS [32,33,34,35,36]. The mentioned miRNAs are involved in the regulation of glucose and lipid metabolism signaling pathways, inflammatory processes, atherogenesis and functioning of the cardiovascular system [37], i.e., processes the disturbances of which determine the development of MetS [2]. Bioinformatic analysis of data on the function of individual miRNAs indicates that their participation in the regulation of signaling pathways is multidirectional and often interconnected [37]. The miRNAs we have selected take part in the control of metabolism by regulating, e.g., glucagon and insulin signaling pathways, as well as mTOR (mammalian target of rapamycin), PI3K-AKT/PKB (phosphatidylinositol 3′-kinase/protein kinase B), AMPK (AMP-activated protein kinase) signaling pathways (miR-15a-5p, miR-23-3p, miR-223-3p), affecting glucose and lipid homeostasis, but also controlling age and hyperglycemia-related complications, such as inflammation, atherosclerosis, thrombogenesis, vascular dysfunction and remodeling (miR-21-5p, miR-223-3p). miR-15a-5p, miR-23-3p and miR-223-3p are involved in the regulation of signaling pathways related to endothelial function as well as anti- and pro-atherogenesis, while miR-23a-3p also controls the level of electrolytes and fluids, indirectly participating in the regulation of blood pressure. Vascular smooth muscle contraction is regulated by the cGMP-PKG (cGMP-dependent protein kinase G) signaling pathway, in which miR-197-3p is involved. Angiogenesis is regulated, among others, by TGF-β (transforming growth factor-β), RAP1 GTPase and EGFR (epidermal growth factor receptor) tyrosine kinase inhibitor resistance signaling pathways, in which miR-15a-5p, miR-21-5p, miR-23-3p, miR-146a-5p and miR-223-3p have regulatory functions. The multidirectional effects associated with MetS also depend on the regulation of the HIF-1 (hypoxia-inducible factor 1) signaling pathway mediated by miR-23-3p and miR-223-3p. As a result of this signaling pathway, the expression of many genes for proteins of anaerobic glucose metabolism and those affecting the proper tone of blood vessels, angiogenesis (vascular endothelial growth factor), inflammatory processes and above all, those responsible for iron metabolism and erythropoiesis (erythropoietin) are regulated. In turn, the FOXO (forkhead box O transcription factor) signaling pathway, which is regulated by miR-15a-5p, miR-21-5p, miR-23-3p and miR-223-3p, affects the expression of genes for proteins involved in the process of glycolysis and gluconeogenesis, but also in increasing the antioxidant capacity (catalase and superoxide dismutase) and immune response. Signaling pathways involved in the control of inflammation, with the regulatory participation of the miRNAs profile selected by us, are also: MAPK (mitogen-activated protein kinase) signaling pathway (miR-15a-5p, miR-21-5p, miR-146a-5p), NF-κB (nuclear factor kappa B) and IL-17 (interleukin 17 family) signaling pathways (miR-146a-5p), as well as the T-cell receptor signaling pathway (miR-223-3p). miR-146-5p participates in the regulation of heat release as a result of increased fatty acid oxidation induced by excessive exposure to cold [37]. Due to the scope of action, dysregulation of the expression of the proposed RNAs profiles (up- or down-regulation) may affect the formation and development of the metabolic syndrome and related complications [32,33,34,35,36,37].

Earlier publications described the down-regulation of circulating miR-15a-5p [32] and miR-21-5p [38] and the up-regulation of circulating miR-197-3p and miR-23a-3p in people with MetS [33]. In the case of pro-inflammatory miR-146a-5p, there was no difference in expression in menopausal women with MetS compared to healthy women of comparable age [38], but up-regulation was found in obese women without MetS compared to a lean 50-year-old [39]. To the best of our knowledge, the expression of circulatory miR-223-3p has not been studied within the context of MetS so far, but miR-223-3p was lower in both overweight and obese subjects compared to the normal-weight control [36]. In both cases, after a 3-month period of increasing energy expenditure through lifestyle changes consisting in the implementation of mixed aerobic and endurance training or aerobic training in combination with a low-fat diet and reducing caloric intake, it resulted in down-regulation regarding the expression of circulating miR-146a-5p [39] and the up-regulation of miR-223-3p, respectively [36]. Changes in the expression of selected circulating miRNAs may be markers of MetS and related dysfunctions, as well as markers of therapeutic effects.

The aim of our study is to: (1) determine which of the proposed miRNA profiles (miR-15a-5p, miR-21-5p, miR-23a-3p, miR-146a-5p, miR-197-3p, miR-223-3p) can be MetS markers; (2) indicate the direction of changes in the expression of selected miRNAs as a result of WBC in postmenopausal women with MetS, in relation to healthy women of comparable age and healthy young women, depending on the number of exposures, i.e., 10 and 20.

It should be emphasized that research on the role of miRNAs in the regulation of metabolism among people with MetS is scarce, and to the best of our knowledge, the impact of WBC on changes in miRNA expression has not been studied so far.

We pose the following hypotheses: (1) In postmenopausal women with MetS, up-regulation occurs in miR-23a-3p, miR-146a-5p and miR-197-3p expression, and simultaneous down-regulation in miR-15a-5p, miR-21-5p and miR-223-3p expression, compared to healthy women at the same age and young women without MetS; (2) changes in the expression of selected microRNAs (miR-15a-5p, miR-21-5p, miR-23a-3p, miR-146a-5p, miR-197-3p, miR-223-3p) occur after applying 20 WBC sessions, and they are greater in postmenopausal women with MetS, compared to healthy women at a similar age and young, healthy women.

## 2. Materials and Methods

### 2.1. Study Design

For the project, 109 volunteers signed up to take part in the trial. The study involved 55 Caucasian women of different ages, performing low or moderate physical activity [40], and for whom there were no contraindications to the use of WBC [17].

The studied women comprised 3 groups:

- H-20—young healthy women, n = 19;
- H-60—healthy postmenopausal women, n = 18;
- M-60—postmenopausal women diagnosed with MetS, n = 18.

**Figure 1:**
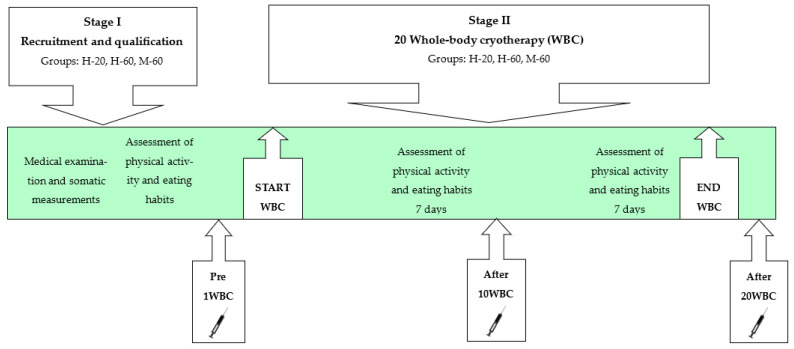
Scheme of the study.

Each subject underwent a series of 20 WBC procedures, which were performed in the afternoon. In addition, miRNA expression (miR-15a-5p, miR-21-5p, miR-23a-3p, miR-146a-5p, miR-197-3p, miR-223-3p) in plasma-suspended peripheral blood mononuclear cells (PBMC) has been determined in a fasting state: before 1 WBC, after 10 WBCs and after 20 WBC treatments (Figure 1, Table 1).

The study was conducted in accordance with the Declaration of Helsinki. The methodology of the study was approved by the Bioethical Committee of the Regional Medical Chamber (284/KBL/OIL/2020, 18 December 2020). 

Postmenopausal women were included in the study due to the higher incidence of MetS in this population group. Although there was no race-specific eligibility requirement, only Caucasians applied for the project, which is probably due to the small proportion of other races in the Polish population where the study was conducted.

Participants were informed verbally and in writing about the purpose and course of the research, about potential risks and benefits, and that they could resign from participating in the research at any stage of its implementation without consequences. All participants provided their written, informed consent to participate in the study.

### 2.2. Qualification of Participants

Young, healthy women (aged 18–25) with a regular menstrual cycle and postmenopausal women aged 55–70 who declared no menstruation for at least 12 months, including healthy individuals and those with MetS, were qualified for the study.

#### 2.2.1. Medical Examination

The volunteers, meeting the above conditions, underwent medical qualification, including medical history, physical examination, blood count, biochemical blood tests (fasting blood glucose (FBG), glycated hemoglobin, lipid profile), assessment of body composition using bioelectrical impedance analysis (BIA), measurement of systolic (SPB) and diastolic (DBP) blood pressure and electrocardiogram examination (ECG).

During the medical examination, each volunteer was diagnosed with MetS according to the NCEP-ATP III criteria [1].

The condition for diagnosing MetS was meeting at least 3 of the following criteria:

- WC (waist circumference) > 88 cm;
- FBG > 5.6 mmol/L;
- HDL-C < 1.3 mmol/L;
- TG > 1.7 mmol/L;
- SBP ≥ 130 mmHg or DBP ≥ 85 mmHg or antihypertensive therapy [1].

After medical qualification, people with any medical contraindications to the use of WBC were excluded from the study [17,41], including BP above 150/90 mmHg [42] and chronically ill people (except MetS in the M-60 group), taking permanent medication (except for antihypertensive therapy in the M-60 group), people declaring smoking, abusing alcohol, other stimulants, as well as people who had already used WBC or other treatments that may affect metabolism, e.g., vibrotherapy, in the last 6 months, sauna and others [43,44,45,46].

#### 2.2.2. Assessment of Physical Activity and Eating Habits

Following a medical examination, the qualified volunteers completed the International Physical Activity Questionnaire (IPAQ)—Polish version [40]. The IPAQ includes questions about physical activity (PA) related to everyday life, professional work and leisure, performed in the 7 days preceding completion of the questionnaire. During a direct meeting with the researcher, the volunteers were instructed on how to fill in the IPAQ correctly. As a result of the IPAQ analysis, the PA of the volunteers was assessed in 1 of 3 categories: high, moderate or low [40].

The eating habits of volunteers were also assessed, who were asked to fill in a daily (for 7 days) food diary, taking the number, time and composition of meals consumed as well as the weight of individual products into account. The portion size and weight of individual products were determined on the basis of a photo album of products and dishes [47]. The average daily supply of energy and individual nutrients was calculated (Dieta6.0, Institute of Food and Nutrition, Warsaw, Poland). The menus were assessed in relation to nutritional standards for the Polish population [48].

People whose PA was high [40] and those using a mixed diet deviating from the recommendations in terms of composition and calorific value [48] were excluded from the study, as well as people using specific diets (vegetarian, vegan, diabetic, low-calorie) or dietary supplements that may affect metabolic rate.

The persons who finally qualified for the study were asked to maintain their previous physical activity as well as diet and not to use any wellness treatments during the WBC period, which was verified by repeating the questionnaire twice (in the 2nd and 4th weeks of the study). In Figure 1, the scheme of the study is shown.

#### 2.2.3. Participants

Inclusion criteria were met by 42 out of 79 postmenopausal women, 20 of whom were diagnosed with MetS. Among 30 young volunteers, 28 met the inclusion criteria. During the period of applying WBC treatments, 15 people resigned, including 13 for organizational and 2 people for health reasons.

The entire study program was completed by 55 volunteers, including 18 healthy (H-60) postmenopausal women (60.28 ± 3.63 years), 18 postmenopausal women (61.33 ± 4.01 years) with MetS (M-60) and 19 young (21.00 ± 1.63 years), healthy women, constituting the comparative group (H-20).

### 2.3. Somatic Measurements and Body Composition Assessment

Body height (BH) was measured using a stadiometer (Seca 217, Hamburg, Germany) to the nearest 1 mm. Body mass (BM), body fat percentage (BF) and lean body mass (LBM) were determined while standing in underwear using the BIA measurement (Jawon IOI-353 Body Composition Analyzer, Gyeongsa, Korea).

Waist circumference (WC) was measured with to the nearest 1 mm in a standing position during the final phase of quiet exhalation, in the narrowest place between the lower edge of the costal arches and the highest point of the iliac crests, placing an anthropometric tape (Seca 201, Hamburg, Germany) perpendicular to the vertical axis of the body.

The participants’ characteristics of somatic structure, divided into groups, are presented in Table 2.

### 2.4. Whole-Body Cryotherapy

The volunteers underwent 20 WBC procedures. Each treatment consisted of a 30 s exposure at −60 °C (in the vestibule of the cryochamber). Then, the volunteers went through an internal door to the directly connected cryochamber proper, where they stayed for 3 min at −120 °C (liquid nitrogen-cooled cryochamber Bamet KN-1, Bamet, Wielka Wieś, Poland). WBC treatments were performed daily at a certified medical facility, i.e., at the Małopolska Cryotherapy Centre in Kraków, in the afternoon (3.00 p.m.–5.00 p.m.), on weekdays (Monday–Friday), in 4 successive series, separated by 2-day breaks (Saturday–Sunday).

The chamber was equipped with an automatic temperature monitoring and air-drying system in the rooms. The oxygen content in the air in the cryo-chamber was kept at a constant level, adequate for the state of normoxia (21–22%) and constantly monitored by 2 independent oxygen probes (EurOx.O2 G/E, Kraków, Poland). In order to ensure safety, both the chamber proper and its vestibule were equipped with alarm buttons and mechanical levers enabling the immediate opening of the door from the inside by the user, as well as an audio-visual system and thermal windows in the chamber door allowing contact with the subjects.

Participants were informed how to prepare for the WBC procedure, how to behave in the cryochamber, and the procedures to be followed in the event of a sudden need to leave the cryochamber were also explained.

The women were instructed not to apply cosmetics to the skin immediately before the procedure, not to depilate or perform activities that may increase the intensity of perspiration, e.g., not to exercise, to dry the skin thoroughly to remove sweat, the presence of which could cause frostbite. Before starting the procedure, jewelry, glasses and contact lenses had to be removed.

The clothing required during the procedures was a sleeveless cotton top and shorts that did not cause pressure on the skin, without metal elements. A hat or headband covering the auricles was put on, thick gloves, the nose and mouth were protected with a surgical mask having an additional layer of gauze, and the ankle and knee joints were covered with woolen socks and protectors. Clogs with wooden soles were used as shoes.

Treatments were performed under the supervision of qualified physiotherapists. SBP and DBP were measured before each procedure. In no case did the measurement result exceed 150/90 mmHg, which was a prerequisite for the procedure [42].

A maximum of 4 people participated in the procedure at a time. During the procedure, it was necessary to walk slowly in a circle, one participant after the other, maintaining calm breathing through the nose and exhaling through the mouth. The direction of the march was changed by a sound signal. Women were asked not to adjust their clothes or touch exposed body parts during the procedure.

According to the International Classification of Medical Procedures (ICD-9), WBC belongs to physiotherapy measures (93.3950). In Poland, WBC treatments are medical services that are used for analgesic, anti-edematous and anti-inflammatory action before kinesiotherapy. These are treatments reimbursed by the National Health Fund (13/2019/DSOZ)—10 treatments performed daily on working days, once every 6 months [49].

### 2.5. Blood Collection

Blood was collected from the venous vessels in the elbow 3 times, i.e., before 1 WBC (pre-1 WBC) and the day following 10 WBCs (post-10 WBCs) and after 20 WBCs (post-20 WBCs), using a vacuum system (Becton Dickinson, Franklin Lakes, NJ, USA). Blood was collected on an empty stomach, between 6:00 a.m. and 8:30 a.m., after approximately 8 h of sleep; the last light meal was eaten 2 h before bedtime. Each time, the blood was collected in a seated position after 5 min of rest.

For miRNA expression analysis, blood was collected into 4 mL Vacutainer BD CPT™ tubes containing sodium citrate and FICOLL™, enabling a one-step, standardized method for the isolation of PBMC—lymphocytes and monocytes from whole blood. In addition, blood was collected into tubes containing EDTA with a glycolysis inhibitor (5 mg NaF and 4 mg potassium oxalate/2 mL of blood) for plasma fasting blood glucose determinations and into tubes with a clotting activator for serum separation in lipid profile determinations. The blood was centrifuged for 15 min (relative centrifugal force: RCF 1000× *g*) using the MPW-351R centrifuge (MPW Med. Instruments, Warsaw, Poland). For plasma and PBMC separation, blood was centrifuged immediately after collection at 4 °C and 21 °C, respectively, while for serum separation, samples were centrifuged at 4 °C after clot formation (approximately 20 min) at 21 °C. The biological material was stored at −80 ± 5 °C until analysis (ZLN-UT 300 PREM, POL-EKO-APARATURA, Wodzisław Śląski, Poland).

### 2.6. miRNA Expression Analysis

The expression of 6 miRNAs was determined: miR-15a-5p, miR-21-5p, miR-23a-3p, miR-146a-5p, miR-197-3p, miR-223-3p, which were selected on the basis of analyzing the results of scientific research [32,33,34,35,36] and bioinformatic tools [37], being important for the regulation of signaling pathways of glucose homeostasis, lipid metabolism and vasomotor activity (Table 1), the disorders of which lead to the development of MetS.

#### 2.6.1. miRNA Isolation and Quality Control of RNA

miRNA was extracted from plasma-suspended PBMCs using a commercial column-based system following the manufacturer’s instructions (MicroRNA Concentrator, A&A Biotechnology, Gdańsk, Poland). Briefly, plasma-suspended PBMCs were thawed on ice and centrifuged at 3000× *g* for 5 min at 4 °C in a microcentrifuge. Then, 200 µL of plasma-suspended PBMCs was transferred to a new microcentrifuge tube, and 750 µL of Fenozol Plus was added. The two-column isolation system allowed to separate long and short RNA fractions. miRNA was eluted by adding 30 µL of ultrapure DNase/RNase-free distilled water to the membrane of the spin column and next carrying out incubation for 3 min before centrifugation at 15,000× *g* for 1 min at room temperature. The RNA was stored at −80 ± 5 °C. The concentration and purity of RNA were measured at 260, 280 and 230 nm using the Spark spectrophotometer with NanoQuant plate (Tecan, Männedorf, Switzerland). A 260:230 ratio greater than 1.7 and a 260:280 ratio greater than 2.0 indicates highly pure RNA.

#### 2.6.2. Reverse Transcription

RNA was reverse-transcribed using the high-capacity cDNA synthesis kit (TaqMan^®^ MicroRNA Reverse transcription Kit Catalogue No. 4366596, Thermo Fisher Scientific, Waltham, MA, USA) and miRNA primers supplied with the TaqMan^®^ miRNA assay. The thermal cycler was set at 16 °C for 30 min, 42 °C for 30 min and 85 °C for 5 min. Then, the temperature was lowered to 4 °C, and the run was stopped. Complementary DNA (cDNA) was stored at −20 ± 2 °C until performing real-time qPCR.

#### 2.6.3. Real-Time Quantitative PCR

Real-time qPCR was conducted on Applied Biosystems TaqMan^®^ TaqMan Fast Advanced Master Mix (Cat. No. 444557, Thermo Fisher Scientific, Waltham, MA, USA), using specific primers supplied by TaqMan^®^ MicroRNA Assays (Cat. No. 4427975, Thermo Fisher Scientific, Waltham, MA, USA): hsa-miR-15a-5p, ID:000389; hsa-miR-21-5p, ID:000397; hsa-miR-23a-3p, ID:000399; hsa-miR-146a-5p, ID:000468; hsa-miR-197-3p, ID:000497; hsa-miR-223-3p, ID:002295.

Negative control samples were added to rule out contamination. RNU6B (ID:001093) was used as the endogenous reference for normalization. All samples were run in duplicates. Real-time qPCR steps included an initial cycle at 95 °C for 10 min, followed by 40 cycles at 95 °C for 15 s, and finally, the temperature was lowered to 60 °C for 1 min. Fluorescent signals from each sample were collected at the endpoint of every cycle. The resulting cycle threshold (Ct) values of the tested samples were determined. The fold change in miRNA expressions was calculated using the relative quantification (RQ) method, where ΔCt sample = CtmiRNA target − CtHK, ΔΔCt = ΔCt sample − average ΔCt non-infected group and RQ = 2 − ΔΔCt. Fold-change values greater than 1 indicate up-regulated expression, while fold-change values less than 1 indicate down-regulated expression [50].

### 2.7. Fasting Blood Glucose and Lipid Profile Analysis

Glucose and lipid profile components were measured via the enzymatic method using reagent kits dedicated to the Cobas c701/702 biochemical analyzer (Roche Diagnostics International Ltd., Rotkreuz, Switzerland), i.e., GLUC3 (detection range 2–750 mg/dL), CHOL2 (detection range 3.86–800 mg/dL), HDLC3 (detection range 3–120 mg/dL), TRIGL (detection range: 8.85–885 mg/dL). The LDL-C concentration was calculated from the formula:

LDL-C = TG − (HDL-C + TG/5).(1)

### 2.8. Statistical Analysis

The distribution of results for the analyzed variables was checked with the Shapiro–Wilk test and the equality of variances using Levene’s test. For single measurements, the significance of inter-group differences was assessed via tests for independent samples (Student’s *t*-test for normally distributed variables or non-parametric Mann–Whitney U test for non-normally distributed variables).

Comparing the impact of WBC treatments on changes in the analyzed variables among the compared groups, analysis of variance with repeated measures (ANOVA) was used, examining the impact of the main factors, i.e., Group (H-20, H-60, M-60), Treatment (WBC) and Group × WBC interaction. If a significant influence of the main factors was found, post hoc analysis was performed using the LSD Fisher test.

Effect sizes for ANOVA analysis were calculated using partial eta squared (η^2^) and interpreted as 0.010–0.059 = small, 0.060–0.139 = medium, ≥0.14 = large.

For changes in the level of specific miRNA after WBC, confidence intervals were determined (95% CI). 

Correlation coefficients were calculated using Spearman’s test. The following correlation assessment was adopted depending on the value of the correlation coefficient—*r*: no correlation if *r* ≤ 0.19, low correlation if 0.20 ≤ *r* ≤ 0.39, moderate correlation if 0.40 ≤ *r* ≤ 0.59, moderately high correlation if 0.60 ≤ *r* ≤ 0.79 and high correlation if *r* ≥ 0.8.

Statistical significance of differences was assumed at *p* < 0.05.

The STATISTICA 13.3 package (StatSoft, Inc., Tulsa, OK, USA) was used for all calculations.

### 2.9. Bioinformatic Analysis

The indicated profile of miRNAs associated with disorders in MetS and the significant signaling pathways controlled by them were selected using the DIANA-miRPath v4.0 database. The following search conditions were used (Targets resource: TarBasev8.0; Terms/Pathways resource: KEGG (Kyoto Encyclopedia of Genes and Genomes); Species: Homo sapiens) [37]. Data are summarized in Table 1.

## 3. Results

### 3.1. Characteristics of the Study Participants

#### 3.1.1. Somatic Build

The M-60 group, compared to H-60 and H-20, demonstrated higher BM (*p* < 0.01), Waist-to-height ratio (WHtR) (*p* < 0.01), %BF (*p* < 0.01) and BMI (*p* < 0.01), but also larger LBM compared to H-60 (*p* = 0.02), with comparable LBM to H-20 (*p* = 0.82). The H-60 and H-20 groups exhibited similar BM (*p* = 0.61), while WHtR (*p* < 0.01), %BF (*p* < 0.01), and BMI (*p* = 0.01) were higher and LBM lower (*p* = 0.01) in the H-60 group compared to H-20 (Table 2).

In the M-60 group (n = 18), only 1 person was characterized by normal BMI; 9 people were overweight, 5 people were in obesity class I, 1 person was in obesity class II, and 2 people were in obesity class III. There were no obese individuals in the H-60 and H-20 groups. In the H-60 group, 7 people were of normal body mass, while 11 people were overweight. In the H-20 group, 13 people demonstrated normal body mass, 5 people were overweight, and 1 was underweight (Table 2).

#### 3.1.2. Diagnostics in Metabolic Syndrome

Among the diagnostic criteria of MetS in the M-60 group, compared to H-60 and H-20, higher WC (*p* < 0.01), TG (*p* < 0.05) and FBG (*p* < 0.01) were found. SBP and DBP did not differ between the M-60 and H-60 groups (*p* > 0.05), but HDL-C was lower (*p* = 0.04), while SBP (*p* = 0.02) and DBP (*p* < 0.01) were higher in the M-60 group compared to H-20. The H-60 group demonstrated greater WC (*p* = 0.01), TG (*p* = 0.01), FBG (*p* = 0.02) and DBP (*p* = 0.02) than H-20 (Table 3).

In the M-60 group, among the MetS criteria adopted by the NCEP-ATP III [1,2], the most frequently repeated disorders were abdominal obesity (in 88.9% of individuals) and hyperglycemia (in 83.3% of individuals). In the M-60 group, 12 subjects (66.7%) met 3 criteria, while 6 (33.3%) met 4 MetS diagnostic criteria. Also, in the groups of healthy women (H-60 and H-20), disorders being risk factors for the development of MetS were noted. Detailed results according to the group are presented in Table 4.

#### 3.1.3. Blood Morphology

Blood count results are shown in Table 5.

#### 3.1.4. Other Metabolic Markers

The compared groups differed significantly (*p* < 0.05) in the percentage of glycated hemoglobin (HbA_1c_), TyG, indices of atherogenesis (atherogenic index of plasma AIP, Castelli index II CRI-II), index of lipid product accumulation (LAP) and index of visceral adipose tissue (VAI), which assumed values in ascending order for groups H-20, H-60 and M-60. T-CHOL, LDL-C and Castelli index I (CRI-I) atherogenic index were comparable in groups M-60 and H-60 (*p* > 0.05) and higher in both groups compared to H-20 (*p* < 0.01) (Table 6).

### 3.2. Expression of Selected miRNAs 

#### 3.2.1. Group Comparison

Significant inter-group differences (ANOVA Group, large effect size) were found in the expression of all analyzed miRs: miR-15a-5p (η^2^ = 0.43, *p* < 0.01), miR-21-5p (η^2^ = 0.48, *p* < 0.01), miR-23a-3p (η^2^ = 0.53, *p* < 0.01), miR-146a-5p (η^2^ = 0.44, *p* < 0.01), miR-197-3p (η^2^ = 0.74, *p* < 0.01) and miR-223-3p (η^2^ = 0.49, *p* < 0.01) (Table 7).

**Figure 2:**
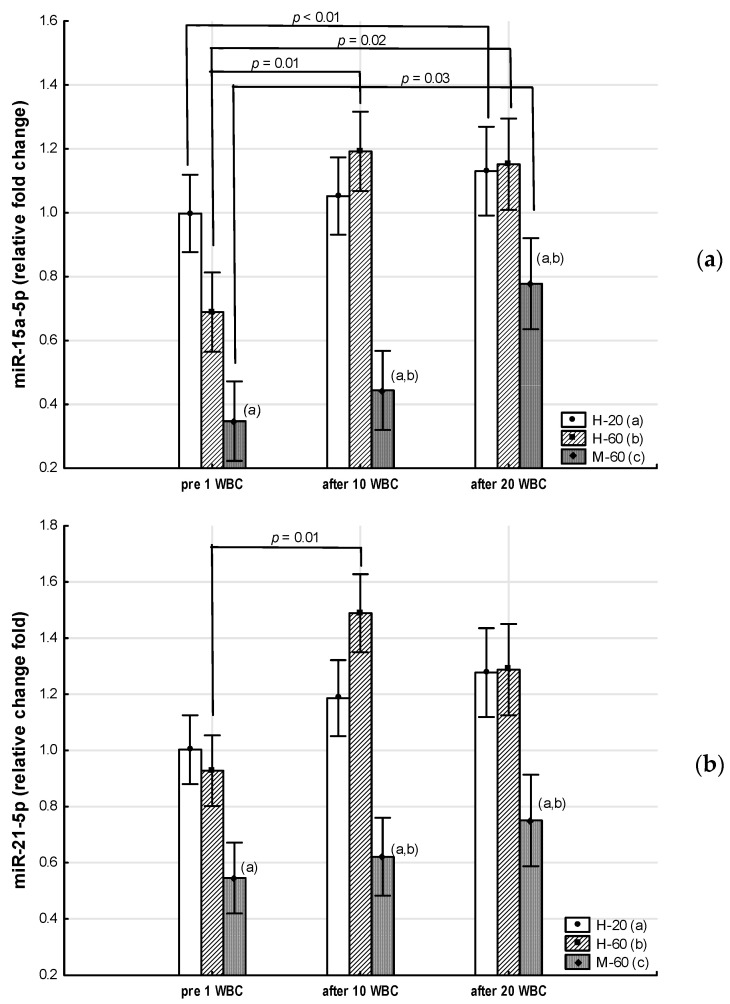

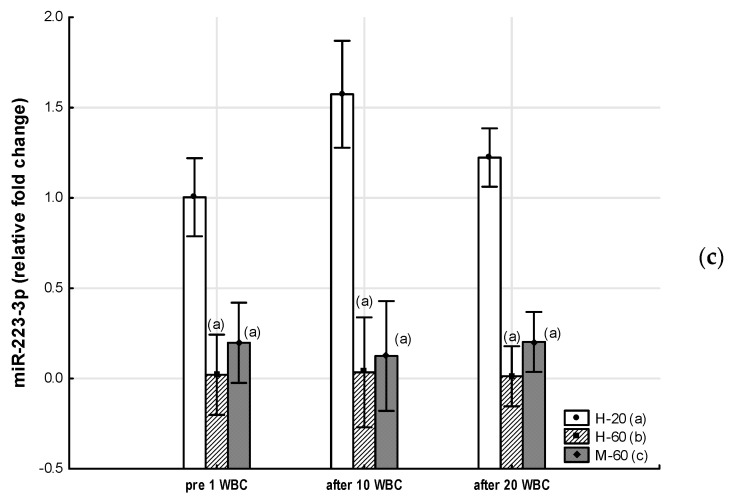
Changes in miR expression as a result of whole-body cryotherapy (WBC) treatments, the expression of which was previously down-regulated (*p* < 0.05) in postmenopausal women with metabolic syndrome (M-60) and/or in healthy post-menopausal women (H-60) compared to young women (H-20): (**a**) miR-15a-5p; (**b**) miR-21-5p; (**c**) miR-223-3pMarkers next to the bars: statistically significant differences (*p* < 0.05): (a) H-60 or M-60 compared to H-20; (b) M-60 compared to H-60.

**Figure 3:**
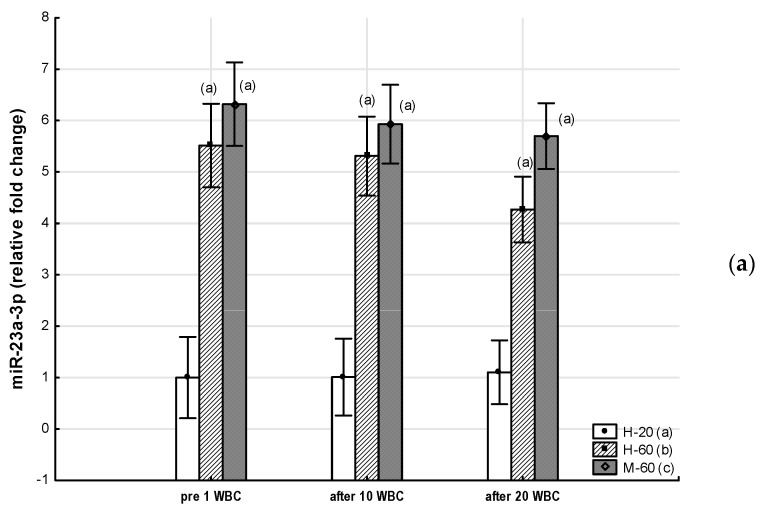

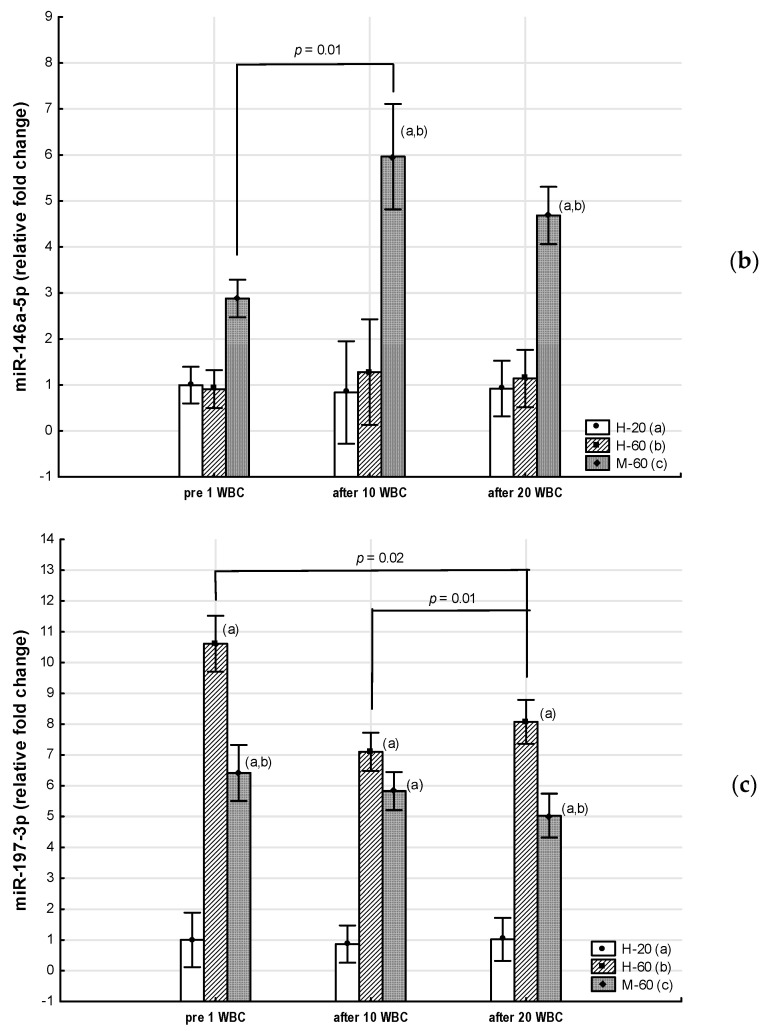
Changes in miR expression as a result of whole-body cryotherapy (WBC) treatments, the expression of which was previously up-regulated (*p* < 0.05) in postmenopausal women with metabolic syndrome (M-60) and/or in healthy postmenopausal women (H-60) compared to young women (H-20): (**a**) miR-23a-3p; (**b**) miR-146a-5p; (**c**) miR-197-3p. Markers next to the bars: statistically significant differences (*p* < 0.05): (a) H-60 or M-60 compared to H-20; (b) M-60 compared to H-60.

Throughout the period of WBC use, the M-60 and H-60 groups were characterized by significantly higher (*p* < 0.05) expression of miR-23a-3p and miR-197-3p, as well as significantly lower (*p* < 0.05) expression of miR-223-3p compared to the H-20 group. After 10 WBCs and 20 WBCs, the expression of miR-197-3p in the M-60 group was lower compared to H-60 (*p* < 0.05). miR-15a-5p and miR-21-5p expression were significantly lower (*p* < 0.05) in the M-60 group compared to H-20 and after 10 WBCs and 20 WBCs, also compared to H-60 (*p* < 0.05). The expression of miR-146a-5p after 10 WBCs and 20 WBCs was significantly higher (*p* < 0.05) in the M-60 group compared to H-20 and H-60 (Table 7, Figure 2 and Figure 3).

#### 3.2.2. Effects of Whole-Body Cryotherapy

A significant effect of WBC (ANOVA WBC, medium effect size) was observed on the change in miR-15a-5p expression (η^2^ = 0.09, *p* = 0.01), miR-21-5p (η^2^ = 0.06, *p* = 0.04), and also miR-197-3p (η^2^ = 0.06, *p* = 0.04) (Table 7).

In the M-60 group, miR-15a-5p expression (0.43: 95%CI 0.14, 0.72) increased significantly (post hoc *p* = 0.03) after 20 WBCs, but miR-146a-5p expression increased significantly (*p* = 0.01) after 10 WBCs (Table 7).

In the H-60 group, after 10 WBCs and 20 WBCs, miR-15a-5p expression significantly increased (0.50: 95%CI 0.17, 0.83; post hoc *p* = 0.01 and 0.46: 95%CI 0.09, 0.83; post hoc *p* = 0.02, respectively), while miR-197-3p expression significantly decreased (−4.00: 95%CI −7.53, −0.48; post hoc *p* = 0.01 and −2.90: 95%CI −7.16, 1.37; post hoc *p* = 0.02, respectively), whereas miR-21-5p expression was significantly (post hoc *p* = 0.01) higher only after 10 WBCs (0.56: 95%CI 0.09, 1.03) (Table 7).

In the H-20 group, miR-15a-5p expression significantly increased after 20 WBCs (post hoc *p* < 0.01) (Table 7, Figure 2 and Figure 3).

#### 3.2.3. Inter-Group Comparison Regarding Effects of Whole-Body Cryotherapy

There was no demonstrated effect (*p* > 0.05) regarding the interaction of Group × WBC factors on the level of the analyzed miRs (Table 7).

### 3.3. Metabolic Changes

#### 3.3.1. Group Comparison

Significant intergroup differences (ANOVA Group, large effect size) were found in the concentration of carbohydrate-lipid metabolism markers: FBG (η^2^ = 0.49, *p* < 0.01), T-CHOL (η^2^ = 0.40, *p* < 0.01), LDL-C (η^2^ = 0.39, *p* < 0.01), TG (η^2^ = 0.33, *p* < 0.01), AIP (η^2^ = 0.30, *p* < 0.01) and TyG (η^2^ = 0.47, *p* < 0.01) (Table 8). 

**Figure 4:**
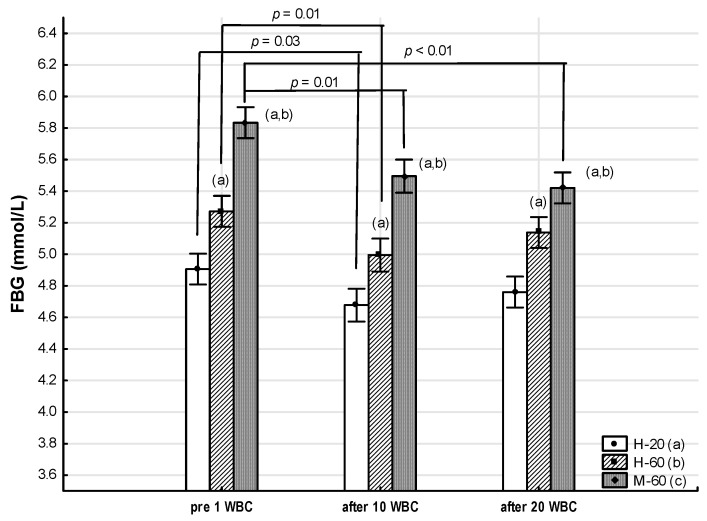
Changes in fasting blood glucose (FBG) as an effect of whole-body cryotherapy (WBC) treatments in postmenopausal women with metabolic syndrome (M-60) and in healthy postmenopausal women (H-60), compared to healthy young women (H-20). Markers next to the bars: statistically significant differences (*p* < 0.05): (a) H-60 or M-60 compared to H-20; (b) M-60 compared to H-60.

Throughout the whole period of WBC use, the M-60 and H-60 groups were characterized by significantly higher (*p* < 0.05) FBG, T-CHOL, LDL-C, TG and TyG values compared to the H-20 group. At the same time, the FBG concentration and the TyG value in the M-60 group for all measurements were significantly higher than in the H-60 group (*p* < 0.05), while the TG concentration was significantly higher in the M-60 group compared to H-60, but only before 1 WBC (*p* < 0.05). The AIP value in each measurement was significantly higher in the M-60 compared to the H-60 and H-20 groups (*p* < 0.05) (Table 8, Figure 4).

#### 3.3.2. Effects of Whole-Body Cryotherapy

A significant effect of WBC (ANOVA WBC) on FBG concentration (η^2^ = 0.20, large effect size, *p* < 0.01) and TyG value (η^2^ = 0.10, medium effect size, *p* = 0.01) was found (Table 8, Figure 4).

In the M-60 group, when using WBC, a significant decrease in FBG concentration was noted both after 10 WBCs (−0.34: 95%CI −0.62, −0.06; post hoc *p* = 0.01) and after 20 WBCs (−0.41: 95%CI −0.70, −0.13; post hoc *p* < 0.01), compared to the value before 1 WBC. At the same time, there was a tendency for TyG values to decrease after 10 WBCs (−0.14: 95%CI −0.28, 0.01; post hoc *p* = 0.07) and after 20WBC (−0.14: 95%CI −0.29, 0.01; post hoc *p* = 0.07) (Table 8).

FBG concentration also significantly decreased after 10 WBCs in the H-60 and H-20 groups (−0.28: 95%CI −0.46, −0.09; post hoc *p* = 0.01 and −0.34: 95%CI −0.62, −0.06; post hoc *p* = 0.03, respectively) (Table 8).

No significant changes in the concentration of T-CHOL, LDL-C, HDL-C, TG or AIP were determined in any of the groups as a result of applying 10 WBCs and 20 WBCs (*p* > 0.05) (Table 8).

#### 3.3.3. Inter-Group Comparison Regarding Effects of Whole-Body Cryotherapy

There was no demonstrated effect (*p* > 0.05) regarding the interaction of Group × WBC factors on the level of carbohydrate-lipid metabolism markers (Table 8).

### 3.4. Correlations between miRNA Expression and Clinically Studied Variables of Metabolic Syndrome as Well as Other Somatic and Metabolic Markers

miRs that were down-regulated in older women (miR-15a-5p, miR-21-5p, miR-223-3p) were correlated negatively with markers of metabolic syndrome as well as other markers of body composition and metabolic disorders, while those that were up-regulated (miR-23a-3p, miR-146a-5p, miR-197-3p) showed a positive correlation. 

**Figure 5:**
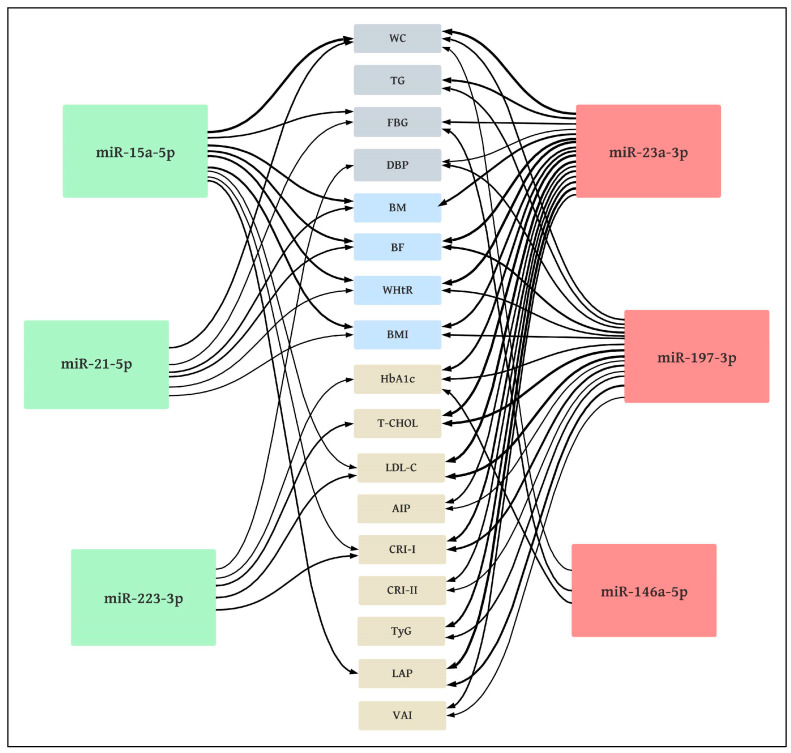
Correlation diagram between miRNA expression and somatic as well as metabolic markers in volunteers. Metabolic syndrome criteria NCEP—ATP III (gray)—WC: waist circumference; TG: triglycerides; FBG: fasting blood glucose; DBP: diastolic blood pressure; body composition markers (blue)—BM: body mass; BF: body fat; WHtR: waist-to-height ratio; BMI: body mass index; other metabolic markers (beige)—HbA_1c_: glycated hemoglobin; T-CHOL: total cholesterol; LDL-C: low-density lipoproteins; AIP: atherogenic index of plasma; CRI-I: Castelli I index; CRI-II: Castelli II index; TyG: triglyceride glucose index; LAP: lipid accumulation product; VAI: visceral adiposity index; down-regulated miRs (green): negative correlations; up-regulated miRs (red): positive correlations; line thickness: correlation coefficient.

These correlations are presented in Table 9 and Figure 5.

The majority of significant correlations were found for miR-23a-3p and miR-197-3p. The strongest correlations (moderately high) were found between miR-23a-3p and BF (*r* = 0.64), WHtR (*r* = 0.60), LAP (*r* = 0.63), and also between miR-197-3p and T-CHOL (*r* = 0.60) as well as LDL-C (*r* = 0.63).

The analyzed miRs demonstrated moderate or low correlations with the MetS criteria. A significant (*p* < 0.05) correlation was found between WC and miR-15a-5p (*r* = −0.59), miR-21-5p (*r* = −0.37), miR-23a-3p (*r* = 0.59), miR-146a-5p (*r* = 0.29) and miR-197-3p (*r* = 0.36); significant correlations (*p* < 0.05) were also noted between TG and miR-23a-3p (*r* = 0.46) and miR-197-3p (*r* = 0.36); more significant (*p* < 0.05) correlations between FBG and miR-15a-5p (*r* = −0.40), miR-21-5p (*r* = −0.28), miR-23a-3p (*r* = 0.37) and miR-146-5p (*r* = 0.37), as well as between DBP and miR-23a-3p (*r* = 0.31), miR-197-3p (*r* = 0.37) and miR-223-3p (*r* = −0.29) were noted. No significant correlations (*p* > 0.05) were observed between the analyzed miRs and HDL-C or SBP.

## 4. Discussion

Disorders of carbohydrate and lipid metabolism in MetS are associated with the endocrine dysfunction of adipocytes and β-pancreatic cells that secrete peptide hormones and/or tissue insensitivity to these hormones. The development of metabolic complications in MetS is associated, among others, with increased concentrations of leptin, asprosin and insulin and decreased concentrations of adiponectin and irisin in the blood. The leptin-to-adiponectin ratio is elevated in MetS subjects and correlates positively with the insulin resistance index. Oxidative stress and chronic inflammation develop [2,21,51,52,53].

So far, the majority of scientists have focused on assessing the concentration of peptide hormones related to carbohydrate-lipid metabolism [16,20,21]. In our research, we concentrated on the study of metabolic changes and those in the expression of miRs, which are involved in the regulation of processes. They are the basis of metabolic and cardiovascular disorders and may play an important role in the development of MetS [26,31,32,33,54,55,56,57]. We assessed the expression of miRNAs in healthy postmenopausal and MetS women compared to healthy young females, as well as their changes as a result of WBC use.

The main finding of our study is that regardless of age, and in older women regardless of MetS, 20 WBCs treatments significantly up-regulate the expression of miR-15a-5p, which correlates negatively with MetS markers (WC, FBG), markers of body composition disorders (BM, BF, WHtR, BMI) and other indices of atherogenesis (LDL-C, CRI-I, LAP). At the same time, in all groups after 10 WBCs, we found a decrease in FBG, which in the group of postmenopausal women with MetS intensified after 20 WBCs, which was accompanied by a downward trend in TyG.

In our research, we have shown that in postmenopausal women with MetS, down-regulation of miR-15a-5p and miR-21-5p expression occur. However, up-regulation of miR-23a-3p and miR-197-3p expression, as well as down-regulation of miR-223-3p expression, take place in postmenopausal women regardless of whether they have metabolic disorders or are healthy.

We also demonstrated that down-regulated miRNAs in postmenopausal women correlated negatively with markers of metabolic syndrome and other markers of body composition as well as metabolic disorders, while those that were up-regulated showed a positive correlation with these indices. 

Among the analyzed miRs profile, the most accurate markers of metabolic disorders turned out to be miR-23a-3p and miR-197-3p, while miR-15a-5p, miR-21-5p and miR-23a-3p showed the most correlations with body composition indices.

One of the miRs we studied is miR-15a-5p, secreted mainly in the pancreas, which is involved in the regulation of pancreatic cell maturation and differentiation, insulin secretion, glucose metabolism, as well as angiogenesis and endothelial dysfunction [58,59,60,61]. Both up- and down-regulation of circulating miR-15a-5p expression in T2DM patients have been demonstrated [29]. Previously, comparing the results of men and women aged approximately 50–60 years, down-regulation of miR-15a-5p expression was shown in healthy women compared to healthy men. At the same time, down-regulation was noted in men and women with MetS compared to healthy individuals [32]. Similar results were also obtained for adolescents, in whom the expression of circulating miR-15a-5p gradually decreased with increasing BMI (normal weight > overweight > obese) [57]. On the other hand, in the group of patients with MetS, without developed diabetes and in those with hypercholesterolemia, up-regulation regarding the expression of circulating miR-197-3p and miR-23a-3p was found. Increased expression of both of these miRs showed a positive correlation with BMI, up-regulation of atherogenic dyslipidemia and vascular inflammation [33].

These results [32,33,57] are consistent with those obtained in our research. In our study, we have shown that circulating miR-197-3p and miR-23a-3p show up-regulation in both healthy postmenopausal women and MetS. For both of these miRs, we found positive correlations with MetS indices such as WC, TG and DBP, and for miR-23a-3p, also with FBG. Similar to the results presented by Ramzan et al. [32], we also observed reduced expression of circulating miR-15a-5p in postmenopausal women with MetS. In our research, we further noted that the down-regulation of miR-15a-5p was more pronounced in subjects with higher FBG and greater visceral obesity.

We have shown that lower expression of circulating miR-15a-5p and higher expression of miR-23a-3p and miR-197-3p are associated with body composition disorders. We found the greatest relationship between the percentage of body fat and WHtR and miR-23a-3p. In other research, it has been shown that WHtR is a valid anthropometric index to diagnose obesity among the elderly and is considered a good indicator in predicting risk factors for cardiovascular diseases, metabolic syndrome and diabetes compared to BMI, WC and other parameters [62]. AWHtR value ≥ 0.5 indicates an increased risk of metabolic diseases [63]. Our results, therefore, indicate the possibility of using miR-23a-3p expression as a marker for the development of metabolic diseases associated with body composition disorders.

Importantly, the increased expression of miR-23a-3p and miR-197-3p, which we found in our research, demonstrated the greatest correlation with markers of metabolic syndrome, atherogenesis and body composition disorders while, at the same time, preceding the manifestation of the disease, so they may be accurate risk markers for the development of MetS.

In our study, we found that miR-223-3p expression is down-regulated in postmenopausal women, regardless of MetS presence. The action of miR-223-3p comprises, among others, the inhibition of genes involved in cholesterol biosynthesis. In addition, miR-223 inhibits the reverse transport of cholesterol from the peripheral cells back to the liver by repressing hepatocyte expression of the scavenger receptor SR-BI, which binds to cholesterol-loaded HDL and transports cholesterol into the liver. The net effect of miR-223 action is to reduce hepatic cholesterol levels [24]. A reduced level of miR-223-3p was observed in obese patients, while after a change in lifestyle, an increase in its level was observed. This indicates the possibility of using miR-223-3p both as a marker of disease and therapy [36]. This is confirmed by our research, in which we showed that reduced expression of miR-223-3p is associated with higher concentrations of T-CHOL, LDL and atherogenesis.

The expression of miR-21-5p, considered a biomarker of atherosclerotic changes in blood vessels, was correlated with the expression of visfatin—an adipokine responsible, inter alia, for the regulation of glucose metabolism in patients with acute coronary syndrome [34]. In our study, decreased expression of miR-21-5p was associated with body composition abnormalities as well as hyperglycemia.

miR-146a expression was shown to be negatively correlated with glycated hemoglobin, insulin resistance, NF-κB mRNA levels and circulating levels of TNFα as well as IL-6 [64]. Reduced expression of miR-146a was associated with the mechanism of developing diabetes and its complications [65]. In cultures of primary adipocytes transfected with miR-146a-5p, a decrease in immune reaction induced by a medium enriched with macrophages was observed [35]. In the context of chronic inflammation accompanying metabolic disorders, this could indicate the benefits of miR-146a-5p expression up-regulation in postmenopausal women with MetS that we found in our study. However, this contradicts our positive correlation between miR-146a-5p and visceral obesity, glycemia and glycated hemoglobin content.

It is known that exposure to cold temperatures can alter the expression of miRs, which are associated with thermoregulatory processes. For example, miR-455 and miR-375 have been noted as associated with the induction of white adipose tissue (WAT) browning in response to cold by up-regulating UCP-1 expression [27,56].

It should be emphasized that research on the role of miRs in the regulation of metabolism among people with MetS is scarce, and to our knowledge, the effect of WBC on changes in the expression of miRs as a potential regulator of translation of peptide hormones related to carbohydrate-lipid metabolism has not been investigated. Therefore, a direct comparison of our results with those obtained by other researchers is impossible.

Menopause is associated with a change in body shape from gynaecoid to android, which is associated with higher cardiometabolic risk and mortality. These changes have been shown to be mediated by a decrease in estrogen and an increase in FSH, which occur in both obese and normal-weight women [66]. In previous studies on postmenopausal women, we indicated that both in healthy individuals and those with MetS, visceral obesity decreases after 20 WBCs, while irisin secretion increases [16]. After 20 WBCs, we found a significant decrease in blood asprosin concentration in the group with MetS but also in the group with hyperglycemia without MetS and in healthy postmenopausal women. A significant decrease was also noted in FBG. We found that the greater the reduction in asprosin level, the lower the level of risk factors for metabolic disorders: e.g., AIP and the leptin/adiponectin index [21]. We also found improvement in glycemia and a decrease in insulin resistance among postmenopausal women with T2DM after 30 WBCs (data in publication). In our current research, we have shown that the use of WBC treatments involving short (3 min) but repeated (20 treatments) impact of cryogenic temperature (minus 120 °C) on the skin induces changes in the expression of miR-15a-5p (up-regulation), regardless of age and the metabolic status of the individuals undergoing therapy. At the same time, we found that miR-15a-5p expression before WBC is down-regulated in postmenopausal women with MetS, and lower levels of expression are associated with body composition disorders, hyperglycemia and atherogenic lipid disorders. Taking these relationships into account, the obtained result clearly indicates a beneficial effect of using WBC in the context of miR-15a-5p-mediated metabolism regulation. The results suggest a beneficial effect of increasing miR-15a-5p expression after 20 WBC treatments. We also obtained a beneficial effect of cryogenic temperatures comprising an increase in the expression of miR-21-5p and a decrease in the expression of miR-197-3p among postmenopausal women without MetS after 10 WBC. Changes in miRs expression were associated with decreased glycemia in all groups as early as after 10 WBCs.

Surprisingly, in our study, miR-146a-5p expression, in contrast to other studies [35,64,65], was up-regulated in postmenopausal women with MetS and was further up-regulated by WBC. This change is associated with the involvement of miR-146a-5p in the regulation of thermogenesis pathways in response to exposure to excessive cold [37].

Due to the high stability in biological fluids, the profile of miRs changes can be postulated as a potential biomarker for diagnosis, prognosis or assessment of disease progression, but also the effects of therapy [30,31]. Designing and translating miR-based therapy from research into clinical applications has great potential [67].

Limitation of the study. In our study, for the first time, beneficial changes are demonstrated in the expression of circulating miRNAs as a result of exposure to cryogenic temperatures in healthy, both young and elderly women, as well as in postmenopausal women with MetS. However, the limitation of our research is the participation of a small number of volunteers and the limited profile of the analyzed miRNAs. It is advisable to extend the scope of research involving more participants of different ages with metabolic disorders, also within the context of differences in reactions according to gender. It would also be interesting to include patients with type 2 diabetes in the study and to determine changes in the expression of miRNAs involved in the regulation of metabolism, pro-oxidative-antioxidant balance, inflammatory processes and endothelial function in response to WBC treatments.

## 5. Conclusions

In our research, it has been shown that miR-23a-3p and miR-197-3p are accurate markers of MetS and MetS risk factors, while miR-15a-5p and miR-23a-3p are precise markers of body composition disorders. WBC is an effective treatment for up-regulating miR-15a-5p and lowering glucose levels in young and postmenopausal women while down-regulating miR-197-3p expression in postmenopausal women. It may be an adjunctive effective treatment method for MetS and hyperglycemia.

## Figures and Tables

**Table 1:** Signaling pathways related to glucose and lipid homeostasis and blood pressure regulation regulated by the analyzed miRNAs (miR-15a-5p, miR-21-5p, 23a-3p, miR-146a-5p, miR-197-3p, miR-223-3p) along with the number of regulated genes.

Signaling Pathway	miRNA Names (Number of Target Genes)
FoxO signaling pathway	miR-15a-5p (31), miR-21-5p (25), miR-23a-3p (24), miR-223-3p (7)
EGFR tyrosine kinase inhibitor resistance	miR-21-5p (14), miR-23a-3p (17), miR-223-3p (6)
MAPK signaling pathway	miR-15a-5p (59), miR-21-5p (39), miR-146a-5p (64)
AMPK signaling pathway	miR-15a-5p (26), miR-23a-3p (22), miR-223-3p (6)
Rap1 signaling pathway	miR-15a-5p (41), miR-23a-3p (33), miR-146a-5p (44)
PI3K-Akt signaling pathway	miR-15a-5p (60), miR-23a-3p (50), miR-223-3p (8)
HIF-1 signaling pathway	miR-23a-3p (22), miR-223-3p (5)
Fluid shear stress and atherosclerosis	miR-15a-5p (28), miR-23a-3p (25), miR-223-3p (4)
mTOR signaling pathway	miR-23a-3p (26), miR-223-3p (6)
cGMP-PKG signaling pathway	miR-197-3p (18)
IL-17 signaling pathway	miR-146a-5p (28)
Insulin signaling pathway	miR-15a-5p (29), miR-23a-3p (24)
Insulin resistance	miR-23a-3p (20), miR-223-3p 95)
AGE-RAGE signaling pathway in diabetic complications	miR-21-5p (18), miR-223-3p (4)
Glucagon signaling pathway	miR-15a-5p (23)
Fatty acid metabolism	miR-15a-5p (16)
TGF-beta signaling pathway	miR-15a-5p (24)
Th17 cell differentiation	miR-223-3p (5)
Thermogenesis	miR-146a-5p (49)
T cell receptor signaling pathway	miR-223-3p (4)
Aldosterone-regulated sodium reabsorption	miR-23a-3p (9)
NF-kappa B signaling pathway	miR-146a-5p (6)

**Table 2:** Somatic build of study participants.

Variable	H-20	H-60	M-60	Statistical Analysis *p*		
	(1)	(2)	(3)	(1–2)	(1–3)	(2–3)
BM (kg)	65.00 ± 9.20	66.32 ± 6.23	77.73 ± 12.18	0.61	<0.01	<0.01
BH (cm)	168.57 ± 5.93	161.39 ± 6.02	160.61 ± 7.37	<0.01	<0.01	0.73
WHtR	0.46 ± 0.05	0.52 ± 0.04	0.59 ± 0.06	<0.01	<0.01	<0.01
BF (%)	27.21 ± 5.32	34.14 ± 3.59	38.71 ± 4.34	<0.01	<0.01	<0.01
LBM (kg)	46.92 ± 3.99	43.57 ± 3.51	47.29 ± 5.47	0.01	0.82	0.02
BMI (kg/m^2^)	22.90 ± 3.19	25.57 ± 2.46	30.56 ± 5.38	0.01	<0.01	<0.01
Obesity classification (BMI)	n (%)	n (%)	n (%)			
underweight (<18.5)	1 (5.3)	0 (0)	0 (0)			
normal weight (18.5–24.9)	13 (68.4)	7 (38.9)	1 (5.6)			
overweight (25.0–29.9)	5 (26.3)	11 (61.1)	9 (50.0)			
obesity class I (30.0–34.9)	0 (0)	0 (0)	5 (27.8)			
obesity class II (35.00–39.9)	0 (0)	0 (0)	1 (5.6)			
obesity class III (>40.0)	0 (0)	0 (0)	2 (11.0)			

Values are means ± SD; BM: body mass, BH: body height, WHtR: waist-to-height ratio, BF: body fat, LBM: lean body mass, BMI: body mass index (BMI = BM (kg)/BH (m)^2^), n (%): number and percentage of persons in the group fulfilling indicated obesity criterion according to BMI; H-20—young, healthy women (n = 19), H-60—healthy postmenopausal women (n = 18), M-60—postmenopausal women diagnosed with metabolic syndrome (n = 18); *p* < 0.05: statistically significant differences (*t*-test or Mann–Whitney U test).

**Table 3:** Medical qualification—metabolic syndrome criteria in the study groups.

Variable	H-20	H-60	M-60	Statistical Analysis *p*		
	(1)	(2)	(3)	(1–2)	(1–3)	(2–3)
MetS Criteria (NCEP—ATP III)						
WC (cm)	69.88 ± 24.25	83.37 ± 4.92	93.77 ± 8.36	0.01	<0.01	<0.01
TG (mmol/L)	0.94 ± 0.33	1.23 ± 0.43	1.61 ± 0.71	0.01	0.01	0.02
HDL-C (mmol/L)	1.71 ± 0.37	1.66 ± 0.41	1.47 ± 0.34	0.69	0.04	0.13
FBG (mmol/L)	4.94 ± 0.43	5.27 ± 0.39	5.83 ± 0.45	0.02	<0.01	<0.01
SBP (mmHg)	113.68 ± 12.68	120.17 ± 16.78	125.83 ± 15.83	0.20	0.02	0.30
DBP (mmHg)	70.53 ± 8.48	77.83 ± 7.69	81.67 ± 6.86	0.02	<0.01	0.12

Values are means ± SD; WC: waist circumference, TG: triglycerides, HDL-C: high-density lipoproteins, FBG: fasting blood glucose, SBP: systolic blood pressure, DBP: diastolic blood pressure; H-20—young, healthy women (n = 19), H-60—healthy postmenopausal women (n = 18), M-60—postmenopausal women diagnosed with metabolic syndrome (n = 18); NCEP-ATP III: National Cholesterol Education Program—Adult Treatment Panel III; *p* < 0.05: statistically significant differences (*t*-test or Mann–Whitney U test).

**Table 4:** Number of people in study groups fulfilling specific metabolic syndrome diagnostic criteria according to assumptions from NCEP-ATP III.

		H-20	H-60	M-60
MetS Diagnostics acc. NCEP—ATP III	MetS Criterion	n (%)	n (%)	n (%)
WC (cm)	>88	2 (10.5)	3 (16.7)	16 (88.9)
TG (mmol/L)	>1.7	1 (5.3)	3 (16.7)	5 (27.8)
HDL-C (mmol/L)	<1.3	4 (21.1)	2 (11.1)	8 (44.4)
FBG (mmol/L)	>5.6	1 (5.3)	3 (16.7)	15 (83.3)
SBP (mmHg)	≥130	2 (10.5)	6 (33.3)	9 (50.0)
DBP (mmHg)	≥85	no one	4 (22.2)	7 (38.9)
Number of fulfilled MetS criteria	0	12 (63.2)	4 (22.2)	no one
	1	4 (21.1)	10 (55.6)	no one
	2	3 (15.8)	4 (22.2)	no one
	3	no one	no one	12 (66.7)
	4	no one	no one	6 (33.3)
	5	no one	no one	no one

NCEP-ATP III: National Cholesterol Education Program—Adult Treatment Panel III, MetS: metabolic syndrome, WC: waist circumference, TG: triglycerides, HDL-C: high-density lipoproteins, FBG: fasting blood glucose, SBP: systolic blood pressure, DBP: diastolic blood pressure; H-20—young, healthy women (n = 19), H-60—healthy postmenopausal women (n = 18), M-60—postmenopausal women diagnosed with metabolic syndrome (n = 18).

**Table 5:** Medical qualification—blood count results in the study groups.

Variable	H-20	H-60	M-60	Statistical Analysis *p*		
	(1)	(2)	(3)	(1–2)	(1–3)	(2–3)
Blood Count						
RBC (10^6^/µL)	4.46 ± 0.24	4.59 ± 0.24	4.62 ± 0.22	0.22	0.04	0.78
HGB (g/dL)	13.23 ± 0.76	13.94 ± 0.55	13.93 ± 0.54	<0.01	<0.01	0.98
HCT (%)	38.29 ± 1.80	41.14 ± 1.62	40.92 ± 1.74	<0.01	<0.01	0.69
PLT (10^3^/µL)	253.0 ± 43.3	254.9 ± 56.6	247.3 ± 79.9	0.91	0.79	0.75
LEUC (10^3^/µL)	5.96 ± 1.10	5.29 ± 1.06	6.16 ± 1.29	0.07	0.61	0.03
NEUT (%)	49.10 ± 7.63	48.36 ± 7.10	50.03 ± 7.67	0.76	0.71	0.50
LYMPH (%)	38.16 ± 6.84	37.56 ± 6.60	37.89 ± 7.59	0.79	0.91	0.89
MONO (%)	10.03 ± 2.24	9.66 ± 2.07	8.13 ± 1.61	0.58	0.01	0.06
EOS (%)	2.05 ± 1.27	3.59 ± 1.77	3.17 ± 1.58	<0.01	<0.01	0.41
BASO (%)	0.66 ± 0.37	0.84 ± 0.59	0.69 ± 0.40	0.24	0.35	0.67

Values are means ± SD; RBC: red blood cells, HGB: hemoglobin, HCT: hematocrit, PLT: platelets, LEUC: leukocytes, NEUT: neutrophils, LYMPH: lymphocytes, MONO: monocytes, EOS: eosinophils, BASO: basophils; H-20—young, healthy women (n = 19), H-60—healthy postmenopausal women (n = 18), M-60—postmenopausal women diagnosed with metabolic syndrome (n = 18); NCEP-ATP III: National Cholesterol Education Program—Adult Treatment Panel III; *p* < 0.05: statistically significant differences (*t*-test or Mann–Whitney U test).

**Table 6:** Medical qualification—other metabolic markers in the study groups.

Variable	H-20	H-60	M-60	Statistical Analysis *p*		
	(1)	(2)	(3)	(1–2)	(1–3)	(2–3)
Other Metabolic Markers						
HbA_1c_ (%)	5.15 ± 0.32	5.65 ± 0.29	5.83 ± 0.28	<0.01	<0.01	0.04
T-CHOL (mmol/L)	4.14 ± 0.81	5.78 ± 0.92	5.52 ± 1.00	<0.01	<0.01	0.42
LDL-C (mmol/L)	2.00 ± 0.65	3.56 ± 0.90	3.32 ± 0.98	<0.01	<0.01	0.45
AIP (log_10_TG/HDL-C)	−0.27 ± 0.20	−0.14 ± 0.20	0.02 ± 0.23	0.04	<0.01	0.04
CRI-I (T-CHOL/HDL-C)	2.48 ± 0.51	3.65 ± 0.92	3.92 ± 0.97	<0.01	<0.01	0.40
CRI-II (LDL-C/HDL-C)	0.60 ± 0.33	0.80 ± 0.37	1.20 ± 0.75	0.03	<0.01	0.04
TyG (lnTG × FBG/2)	8.16 ± 0.31	8.50 ± 0.30	8.85 ± 0.41	<0.01	<0.01	0.01
LAP (WC-58) × TG	18.36 ± 8.16	31.04 ± 11.14	57.24 ± 26.43	<0.01	<0.01	<0.01
VAI	1.09 ± 0.61	1.47 ± 0.66	2.24 ± 1.29	0.08	<0.01	0.03

Values are means ± SD; HbA_1c_: glycated hemoglobin, T-CHOL: total cholesterol, LDL-C: low-density lipoproteins, AIP: atherogenic index of plasma, CRI-I: Castelli I index, CRI-II: Castelli II index, TyG: triglyceride glucose index, VAI: visceral adiposity index (VAI_women_ = [WC/(36.58 + (1.89 × BMI))] × (TG/0.81) × (1.52/HDL-C)), LAP: lipid accumulation product; H-20—young, healthy women (n = 19), H-60—healthy postmenopausal women (n = 18), M-60—postmenopausal women diagnosed with metabolic syndrome (n = 18); NCEP-ATP III: National Cholesterol Education Program—Adult Treatment Panel III; *p* < 0.05: statistically significant differences (*t*-test or Mann–Whitney U test).

**Table 7:** Comparing the expression of selected miRNAs in the groups and changes under the influence of whole-body cryotherapy.

			Mean ± SD		ANOVA				Mean (95% CI)	Post hoc	Mean (95% CI)	Post hoc
Variable (Relative Fold Change)	Group	(1) Pre 1 WBC	(2) After 10 WBCs	(3) After 20 WBCs		Group	WBC	Group × WBC	(2-1) Δ10 WBCs	*p*	(3-1) Δ20 WBCs	*p*
	H-20 (a)	1.00 ± 0.74	1.05 ± 0.79	1.13 ± 0.73	*p*	<0.01	0.01	0.32	0.05 (−0.47; 0.57)	0.77	0.13 (−0.40; 0.67) *	<0.01
miR-15a-5p	H-60 (b)	0.69 ± 0.50	1.19 ± 0.40	1.15 ± 0.43	F	19.47	4.88	1.18	0.50 (0.17; 0.83) *	0.01	0.46 (0.09; 0.83) *	0.02
	M-60 (c)	0.35 ± 0.16 ^a^	0.44 ± 0.16 ^ab^	0.78 ± 0.61 ^ab^	η^2^	0.43	0.09	0.04	0.10 (0.00; 0.19)	0.62	0.43 (0.14; 0.72) *	0.03
	H-20 (a)	1.00 ± 0.62	1.19 ± 0.85	1.28 ± 0.78	*p*	<0.01	0.04	0.58	0.18 (−0.33; 0.69)	0.39	0.27 (−0.30; 0.85)	0.20
miR-21-5p	H-60 (b)	0.93 ± 0.64	1.49 ± 0.48	1.29 ± 0.61	F	23.54	3.23	0.72	0.56 (0.09; 1.03) *	0.01	0.36 (−0.11; 0.83)	0.10
	M-60 (c)	0.55 ± 0.25 ^a^	0.62 ± 0.27 ^ab^	0.75 ± 0.66 ^ab^	η^2^	0.48	0.06	0.03	0.08 (−0.07; 0.22)	0.73	0.21 (−0.15; 0.57)	0.35
	H-20 (a)	1.00 ± 0.63	1.01 ± 0.70	1.10 ± 1.03	*p*	<0.01	0.56	0.87	0.01 (−0.49; 0.51)	0.99	0.11 (−0.40; 0.61)	0.91
miR-23a-3p	H-60 (b)	5.51 ± 5.27 ^a^	5.31 ± 4.78 ^a^	4.27 ± 4.11 ^a^	F	29.91	0.58	0.30	−0.21 (−4.11; 3.69)	0.83	−1.28 (−4.72; 2.16)	0.20
	M-60 (c)	6.32 ± 2.85 ^a^	5.93 ± 3.01 ^a^	5.70 ± 2.12 ^a^	η^2^	0.53	0.01	0.01	−0.40 (−1.75; 0.94)	0.69	−0.64 (−2.50; 1.22)	0.52
	H-20 (a)	1.00 ± 1.45	0.84 ± 0.77	0.92 ± 1.00	*p*	<0.01	0.24	0.31	−0.13 (−0.62; 0.36)	0.89	−0.06 (−0.82; 0.70)	0.95
miR-146a-5p	H-60 (b)	0.91 ± 1.21	1.28 ± 0.99	1.14 ± 2.62	F	20.80	1.46	1.21	0.30 (−0.35; 0.94)	0.74	0.18 (−0.95; 1.32)	0.84
	M-60 (c)	2.88 ± 2.37	5.96 ± 8.42 ^ab^	4.68 ± 3.67 ^ab^	η^2^	0.44	0.03	0.04	2.47 (−0.81; 5.75) *	0.01	1.45 (−0.25; 3.14)	0.11
	H-20 (a)	1.00 ± 0.47	0.87 ± 0.35	1.02 ± 0.58	*p*	<0.01	0.04	0.14	−0.15 (−0.50; 0.19)	0.89	0.02 (−0.42; 0.46)	0.99
miR-197-3p	H-60 (b)	10.61 ± 5.72 ^a^	7.10 ± 3.66 ^a^	8.07 ± 4.89 ^a^	F	74.47	3.42	1.78	−4.00 (−7.53; −0.48) *	0.01	−2.90 (−7.16;1.37) *	0.02
	M-60 (c)	6.41 ± 3.53 ^ab^	5.82 ± 2.77 ^a^	5.03 ± 1.95 ^ab^	η^2^	0.74	0.06	0.06	−0.67 (−2.81; 1.47)	0.58	−1.58 (−3.97; 0.82)	0.19
	H-20 (a)	1.00 ± 1.58	1.57 ± 2.19	1.22 ± 1.18	*p*	<0.01	0.67	0.68	0.29 (−0.41; 1.00)	0.08	0.11 (−0.35; 0.57)	0.50
miR-223-3p	H-60 (b)	0.02 ± 0.03 ^a^	0.03 ± 0.04 ^a^	0.01 ± 0.01 ^a^	F	25.29	0.39	0.58	0.01 (−0.01; 0.02)	0.97	0.00 (−0.01; 0.01)	0.98
	M-60 (c)	0.20 ± 0.25 ^a^	0.12 ± 0.12 ^a^	0.20 ± 0.24 ^a^	η^2^	0.49	0.01	0.02	−0.04 (−0.10; 0.03)	0.83	0.00 (−0.08; 0.09)	0.99

SD: standard deviation, CI: confidence interval, WBC: whole-body cryotherapy, Δ10 WBCs: difference after 10 WBC compared to pre 1 WBC, Δ20 WBC: difference after 20 WBCs compared to pre-1 WBC; statistically significant differences (*p* < 0.05): * compared to values pre 1 WBC, ^a^ H-60 or M-60 compared to H-20; ^b^ H-60 compared to M-60.

**Table 8:** Comparison of carbohydrate-lipid metabolism markers in the groups and changes under the influence of whole-body cryotherapy.

			Mean ± SD		ANOVA				Mean (95% CI)	Post hoc	Mean (95% CI)	Post hoc
Variable	Group	(1) Pre 1 WBC	(2) After 10 WBCs	(3) After 20 WBCs		Group	WBC	Group × WBC	(2-1) Δ10 WBCs	*p*	(3-1) Δ20 WBCs	*p*
	H-20 (a)	4.94 ± 0.43	4.68 ± 0.38	4.77 ± 0.40	*p*	<0.01	<0.01	0.27	−0.23 (−0.40; −0.06) *	0.03	−0.17 (−0.43; 0.08)	0.16
FBG	H-60 (b)	5.27 ± 0.39 ^a^	5.00 ± 0.30 ^a^	5.14 ± 0.39 ^a^	F	24.40	13.06	1.31	−0.28 (−0.46; −0.09) *	0.01	−0.13 (−0.24; −0.03)	0.19
(mmol/L)	M-60 (c)	5.83 ± 0.45 ^ab^	5.50 ± 0.59 ^ab^	5.42 ± 0.45 ^ab^	η^2^	0.49	0.20	0.05	−0.34 (−0.62; −0.06) *	0.01	−0.41 (−0.70; −0.13) *	<0.01
	H-20 (a)	4.14 ± 0.81	4.01 ± 0.85	4.02 ± 0.62	*p*	<0.01	0.27	0.85	−0.12 (−0.27; 0.04)	0.38	−0.15 (−0.36; 0.05)	0.30
T-CHOL	H-60 (b)	5.78 ± 0.92 ^a^	5.69 ± 0.90 ^a^	5.65 ± 1.16 ^a^	F	16.60	1.34	0.34	−0.09 (−0.28; 0.11)	0.44	−0.13 (−0.40; 0.14)	0.25
(mmol/L)	M-60 (c)	5.52 ± 1.00 ^a^	5.58 ± 0.99 ^a^	5.45 ± 1.08 ^a^	η^2^	0.40	0.03	0.01	0.06 (−0.15; 0.27)	0.58	−0.07 (−0.41; 0.28)	0.54
	H-20 (a)	2.00 ± 0.65	1.95 ± 0.73	1.98 ± 0.53	*p*	<0.01	0.64	0.64	−0.03 (−0.18; 0.12)	0.75	−0.06 (−0.18; 0.07)	0.73
LDL-C	H-60 (b)	3.56 ± 0.90 ^a^	3.45 ± 0.89 ^a^	3.41 ± 1.09 ^a^	F	16.06	0.44	0.64	−0.11 (−0.32; 0.10)	0.30	−0.14 (−0.40; 0.12)	0.17
(mmol/L)	M-60 (c)	3.32 ± 0.98 ^a^	3.43 ± 0.99 ^a^	3.34 ± 1.03 ^a^	η^2^	0.39	0.01	0.02	0.11 (−0.09; 0.31)	0.30	0.02 (−0.30; 0.34)	0.86
	H-20 (a)	1.71 ± 0.37	1.69 ± 0.42	1.66 ± 0.36	*p*	0.13	0.60	0.78	−0.03 (−0.11; 0.06)	0.79	−0.04 (−0.14; 0.06)	0.42
HDL-C	H-60 (b)	1.66 ± 0.41	1.70 ± 0.43	1.69 ± 0.45	F	2.14	0.52	0.44	0.04 (−0.04; 0.11)	0.36	0.03 (−0.09; 0.15)	0.45
(mmol/L)	M-60 (c)	1.47 ± 0.34 ^a^	1.48 ± 0.31	1.44 ± 0.33	η^2^	0.08	0.01	0.02	0.02 (−0.05; 0.08)	0.70	−0.03 (−0.10; 0.04)	0.50
	H-20 (a)	0.94 ± 0.33	0.82 ± 0.18	0.84 ± 0.19	*p*	<0.01	0.24	0.96	−0.12 (−0.29; 0.05)	0.27	−0.11 (−0.25; 0.03)	0.34
TG	H-60 (b)	1.23 ± 0.43 ^a^	1.20 ± 0.59 ^a^	1.19 ± 0.51 ^a^	F	12.32	1.46	0.16	−0.03 (−0.26; 0.20)	0.77	−0.04 (−0.35; 0.27)	0.74
(mmol/L)	M-60 (c)	1.61 ± 0.71 ^ab^	1.48 ± 0.51 ^a^	1.48 ± 0.45 ^a^	η^2^	0.33	0.03	0.01	−0.13 (−0.43; 0.16)	0.22	−0.13 (−0.40; 0.14)	0.24
	H-20 (a)	−0.27 ± 0.20	−0.31 ± 0.16	−0.30 ± 0.14	*p*	<0.01	0.26	0.99	−0.04 (−0.13; 0.05)	0.31	−0.03 (−0.10; 0.03)	0.44
AIP	H-60 (b)	−0.14 ± 0.20	−0.17 ± 0.22	−0.17 ± 0.25	F	10.65	1.37	0.03	−0.03 (−0.10; 0.04)	0.43	−0.03 (−0.16; 0.10)	0.47
	M-60 (c)	0.02 ± 0.23 ^ab^	−0.02 ± 0.22 ^ab^	0.00 ± 0.23 ^ab^	η^2^	0.30	0.03	<0.01	−0.04 (−0.12; 0.04)	0.34	−0.02 (−0.09; 0.05)	0.63
	H-20 (a)	8.16 ± 0.31	8.02 ± 0.23	8.04 ± 0.26	*p*	<0.01	0.01	0.98	−0.16 (−0.35; 0.03)	0.07	−0.13 (−0.26; 0.00)	0.10
TyG	H-60 (b)	8.50 ± 0.30 ^a^	8.40 ± 0.37 ^a^	8.42 ± 0.37 ^a^	F	22.11	5.18	0.09	−0.11 (−0.24; 0.03)	0.17	−0.08 (−0.30; 0.14)	0.28
	M-60 (c)	8.85 ± 0.41 ^ab^	8.71 ± 0.41 ^ab^	8.71 ± 0.36 ^ab^	η^2^	0.47	0.10	<0.01	−0.14 (−0.28; 0.01)	0.07	−0.14 (−0.29; 0.01)	0.07

SD: standard deviation, CI: confidence interval, WBC: whole-body cryotherapy, Δ10 WBC: difference after 10 WBCs compared to pre-1WBC, Δ20 WBCs: difference after 20 WBCs compared to pre-1 WBC; FBG: fasting blood glucose, T-CHOL: total cholesterol, LDL-C: LDL-cholesterol, HDL-C: HDL-cholesterol, TG: triglycerides, AIP: atherogenic index of plasma, TyG: triglyceride glucose index; statistically significant differences (*p* < 0.05): * compared to values pre 1WBC, ^a^ H-60 or M-60 compared to H-20; ^b^ H-60 compared to M-60.

**Table 9:** Correlations between relative fold change in miRNAs expression and clinically studied variables of metabolic syndrome as well as other somatic and metabolic markers in volunteers.

Variable	miR-15a-5p	miR-21-5p	miR-23a-3p	miR-146a-5p	miR-197-3p	miR-223-3p
	MetS Criteria (NCEP—ATP III)					
WC	−0.59	−0.37	0.59	0.29	0.36	NS
TG	NS	NS	0.46	NS	0.36	NS
HDL-C	NS	NS	NS	NS	NS	NS
FBG	−0.40	−0.28	0.37	0.37	NS	NS
SBP	NS	NS	NS	NS	NS	NS
DBP	NS	NS	0.31	NS	0.37	−0.29
	Indices of Body Composition					
BM	−0.51	−0.37	0.48	NS	NS	NS
LBM	NS	NS	NS	NS	NS	NS
BF	−0.52	−0.35	0.64	NS	0.48	NS
WHtR	−0.53	−0.33	0.60	NS	0.40	NS
BMI	−0.53	−0.34	0.54	NS	0.37	NS
	Other Metabolic Markers					
HbA_1c_	NS	NS	0.50	0.38	0.37	−0.32
T-CHOL	NS	NS	0.57	NS	0.60	−0.39
LDL-C	−0.27	NS	0.58	NS	0.63	−0.44
AIP	NS	NS	0.39	NS	0.32	NS
CRI-I	−0.29	NS	0.54	NS	0.53	−0.35
CRI-II	NS	NS	0.39	NS	0.32	NS
TyG	NS	NS	0.48	NS	0.39	NS
LAP	−0.39	NS	0.63	NS	0.45	NS
VAI	NS	NS	0.40	NS	0.32	NS

WC: waist circumference, TG: triglycerides; HDL-C: high-density lipoproteins; FBG: fasting blood glucose; SBP: systolic blood pressure; DBP: diastolic blood pressure; BM: body mass; LBM: lean body mass; BF: body fat, WHtR: waist-to-height ratio; BMI: body mass index; HbA_1c_: glycated hemoglobin; T-CHOL: total cholesterol; LDL-C: low-density lipoproteins; AIP: atherogenic index of plasma; CRI-I: Castelli I index; CRI-II: Castelli II index; TyG: triglyceride glucose index; LAP: lipid accumulation product; VAI: visceral adiposity index. NS: not statistically significant (*p* ≥ 0.05).

## Data Availability

The datasets used and/or analyzed during the current study are available from the corresponding author upon reasonable request.
